# Higher resolution in cryo-EM by the combination of macromolecular prior knowledge and image-processing tools

**DOI:** 10.1107/S2052252522006959

**Published:** 2022-08-03

**Authors:** Erney Ramírez-Aportela, Jose M. Carazo, Carlos Oscar S. Sorzano

**Affiliations:** a Biocomputing Unit, National Centre for Biotechnology (CNB CSIC), Darwin 3, Campus Universidad Autónoma de Madrid, Cantoblanco, Madrid 28049, Spain; b Universidad CEU San Pablo, Campus Urb. Montepríncipe, Boadilla del Monte, Madrid 28668, Spain; Chinese Academy of Sciences, China

**Keywords:** deep learning, *RELION*, *deepEMhancer*, prior knowledge, cryo-electron microscopy

## Abstract

Single-particle cryo-electron microscopy has become a powerful technique for the 3D structure determination of biological molecules. The last decade has seen an astonishing development of both hardware and software, and an exponential growth of new structures obtained at medium-high resolution. However, the knowledge accumulated in this field over the years has hardly been utilized as feedback in the reconstruction of new structures. In this context, this article explores the use of the deep-learning approach *deepEMhancer* as a regularizer in the *RELION* refinement process. *deepEMhancer* introduces prior information derived from macromolecular structures, and contributes to noise reduction and signal enhancement, as well as a higher degree of isotropy. These features have a direct effect on image alignment and reduction of overfitting during iterative refinement. The advantages of this combination are demonstrated for several membrane proteins, for which it is especially useful because of their high disorder and flexibility.

## Abstract 2

This article explores the use of the deep learning approach *deepEMhancer* as a regularizer in the *RELION* refinement process. *deepEMhancer* introduces prior information derived from macromolecular structures and contributes to noise reduction and signal enhancement, as well as a higher degree of isotropy, with a direct effect on image alignment and a reduction of overfitting during iterative refinement.

## 1. Introduction

Single-particle analysis of macromolecular structures by electron cryo-microscopy (cryo-EM) has been established as a key technique in structural biology, with the power to reach near-atomic resolutions and explore conformational flexibility. Thousands or even millions of projections of the macromolecule of interest in different orientations (2D images obtained in the microscope) are used to determine its 3D structure. Different algorithms have been developed for the 3D reconstruction process from the images (Grant *et al.*, 2018 ▸; Grigorieff, 2007 ▸; Ludtke *et al.*, 1999 ▸; Punjani *et al.*, 2017 ▸; Scheres, 2012*a* ▸; Sorzano *et al.*, 2018 ▸), in which the orientation and position of the particles in each image are inferred by comparing them with *in silico* projections of a reference map. However, the images acquired at the microscope are extreme­ly noisy, which make the search for the correct angular assignment of the particles a challenge.

One of the most established reconstruction approaches in the field is based on iterative refinement formulated as maximum *a posteriori* (MAP) optimization (Punjani *et al.*, 2017 ▸; Scheres, 2012*a* ▸, 2012*b* ▸), although the practical use of rich priors in these methods is very limited, which we will comment on later. At each iteration, the angular assignment of each particle is updated, while the new maps obtained are modified (by regularization) to suppress noise and thus reduce overfitting. The most commonly used strategy to avoid overfitting is a regularizer in the form of a space-invariant filter, which is applied equally throughout the space and is determined by the Fourier shell correlation (FSC) (Chen *et al.*, 2013 ▸; Harauz & Heel, 1986 ▸; Rosenthal & Henderson, 2003 ▸; Scheres, 2012*a* ▸; Scheres & Chen, 2012 ▸). However, these filters do not consider the spatial heterogeneity of the maps (*i.e.* different regions may have different resolutions), although approaches have been developed to use local filtering after refinement (Cardone *et al.*, 2013 ▸; Kucukelbir *et al.*, 2014 ▸; Vilas *et al.*, 2018 ▸), achieving more reliable results.

Other solutions have been presented with the purpose of mitigating local overfitting. In *cryoSPARC*, the non-uniform refinement algorithm (Punjani *et al.*, 2020 ▸) introduces an adaptive cross-validation regularization which is applied at each iteration of the refinement. In the case of *RELION* (Scheres, 2012*a* ▸), the new external reconstruction functionality (Kimanius *et al.*, 2021 ▸) allows investigation of ways to include the introduction of priors through a script that runs external software to modify the intermediate maps without regularization. This functionality is used by *SIDESPLITTER* (Ramlaul *et al.*, 2020 ▸), which relies on a modified adaptation of the *LAFTER* algorithm (Ramlaul *et al.*, 2019 ▸) to denoise intermediate maps during refinement.

Currently, deep-learning-based methods have a high impact on cryo-EM and are being used in different stages of processing such as denoising (Bepler *et al.*, 2020 ▸), particle picking (Wagner *et al.*, 2019 ▸; Wang *et al.*, 2016 ▸), map reconstruction (Gupta *et al.*, 2021 ▸; Zhong *et al.*, 2020 ▸) or local resolution estimation (Ramírez-Aportela *et al.*, 2019 ▸). Indeed, the idea of using tools based on deep learning to denoise the intermediate maps within the iterative process of density-map refinement has also been raised (Kimanius *et al.*, 2021 ▸), which would allow us to introduce previously acquired knowledge about biological macromolecules to the refinement process. However, this approach was only tested on simulated maps and has not been applied to experimental data.

In this paper we present the first development in which rich, protein-specific, prior information derived from experimental information deposited in public databases is applied to experimental data for alignment. We incorporate this prior information through the use of our recently introduced deep-learning approach *deepEMhancer* (Sanchez-Garcia *et al.*, 2021 ▸), which is applied iteratively within *RELION*. *deepEMhancer* performs a non-linear transformation of the volume that produces a new density map which incorporates EMDB-related prior information resulting in masking/denoising, sharpening effects as well as a higher degree of isotropy. Naturally, this additional information is expected to be especially useful in difficult cases in which parts of the map are affected differently by flexibility/local disorder or local noise, as is typically the case for membrane proteins embedded in lipid bilayers. We would also expect that increased map isotropy would help in extracting the most from samples presenting preferred orientations, rather than a possible exacerbation of this problem during refinement. Our test clearly shows substantial enhancement of the results when compared with standard *RELION* and *SIDESPLITTER*.

## 2. Methods

### 2.1. *deepEMhancer* in *relion_refine*

In order to facilitate better treatment of the signal during the reconstruction of the maps and avoid overfitting, *deepEMhancer* was integrated into the iterative process of *relion_refine*. It was previously shown that *deepEMhancer* boosts the signal of the map (sharpening effect) while at the same time producing a noise reduction effect. Also, *deepEMhancer* incorporates prior information from macromolecules acquired during its deep-learning process. These benefits were further demonstrated in membrane proteins: suppressing most of the signal coming from the lipid layer and enhancing the signal belonging to the protein (Sanchez-Garcia *et al.*, 2021 ▸).

Since version 3.1, an external reconstruction functionality was enabled in the *RELION* refinement program (Kimanius *et al.*, 2021 ▸). When the --external_reconstruct argument is used, *relion_refine* waits while an external program modifies the unregularized half-maps at each iteration. In this work, this new functionality is used by *deepEMhancer* to enhance the signal and remove noise from the intermediate reconstructions. *deepEMhancer* is applied independently to each of the generated half-maps. However, it is only executed in the last iterations, when the process enters the local angular search. For this purpose, the variable *rlnHealpixOrder* in the relion_iter_sampling.star file is monitored at each iteration.

### 2.2. Reconstruction using simulated datasets

Initially, the proposed refinement protocol was studied using simulated data. The first case was based on the structure of β-galactosidase (PDB entry 3j7h; Bartesaghi *et al.*, 2014 ▸). A density map with a sampling rate of 0.637 Å and a box size of 338 × 338 × 338 was derived from the structural model, calling the function *xmipp_volume_from_pdb* (Sorzano *et al.*, 2015 ▸) from the *Xmipp* package (de la Rosa-Trevín *et al.*, 2013 ▸; Strelak *et al.*, 2021 ▸). Using this map, projections were generated in all directions with an angular sampling of 1.5°, for a total of 18 309 projections. Gaussian noise with zero mean and a standard deviation of 150 was added to the set of projections (as shown in Fig. S1 of the supporting information, where images with noise of different standard deviations are presented). The projections were then used for the unmasked ‘3D auto-refinement’ in *RELION* while the initial map was taken as a reference.

The second case tested was based on the structure of the 20S proteasome (PDB entry 6bdf; Campbell *et al.*, 2015 ▸). A similar protocol to the previous one was followed. However, in this case the map was created with a box size of 256 × 256 × 256, a sampling rate of 1.0 Å and the set of projections generated was anisotropic, increasing projections in the cone formed between tilt angles of 0 and 40°. A total of 24 359 projections were generated and processed for refinement. In both cases *RELION* (version 3.1) was used and no solvent-mask was provided.

In a third case, attempting to get closer to a real experiment, 18 309 projections generated from the reconstructed map using the EMPIAR-10391 dataset were added to ‘pure noise’ particles picked over the deposited micrographs. Micrograph CTF estimation was performed using *GCTF* (Zhang, 2016 ▸). *JANNI* (Wagner & Raunser, 2020 ▸) was used to denoise and facilitate picking in areas where there was no particulate matter. For noise particle selection we use the *Xmipp* particle-picking algorithm (Abrishami *et al.*, 2013 ▸). The extracted particles were subjected to two rounds of 2D classification using *cryoSPARC* (Punjani *et al.*, 2017 ▸) to discard particles with macromolecular signal. Finally, 18 309 ‘pure noise’ particles were selected. To study the effect of noise level, the intensity of the selected particles was increased by 5, 10 and 15×, respectively.

### 2.3. Experimental datasets

Three datasets were obtained from *EMPIAR* to test the applicability of *deepEMhancer* within the *RELION* refinement: EMPIAR-10391 (Tan *et al.*, 2020*b* ▸), EMPIAR-10254 (Dang *et al.*, 2019 ▸) and EMPIAR-10420 (Tan *et al.*, 2020*a* ▸). All refinements were computed from particle image stacks, with no further pre-processing. *RELION* (version 3.1) was used and no solvent-mask was provided. The EMDB maps (EMD-21600, EMD-0594 and EMD-21983, respectively), filtered at 20 Å, were used as the initial maps for the refinements. All refinements were carried out through *Scipion* (version 3; de la Rosa-Trevín *et al.*, 2016 ▸).

### 2.4. Implementation details

Based on the new external reconstruction functionality in *relion_refine*, a python script was created to couple *deepEMhancer* in the *RELION* refinement. The python script can be downloaded from *GitHub* (https://github.com/erneyramirez/relion_deepEMhancer_extRec). In addition, *deepEMhancer* is freely available at the author’s *GitHub* site (https://github.com/rsanchezgarc/deepEMhancer) and as an *Xmipp* protocol for *Scipion* (version 3; https://github.com/I2PC/scipion-em-xmipp).

## 3. Results

### 3.1. Test with simulated data

To test the effect of *deepEMhancer* application on un­regularized reconstructions in the angular local search of *RELION*, we initially used simulated data. The first case studied was based on the structure of the β-galactosidase (PDB entry 3j7h). A set of projections was generated based on the simulated map obtained from the atomic model. This set of projections was then used to compare the reconstructions obtained by applying standard *RELION*-only refinement, and then either using *SIDESPLITTER* or the newly proposed method. The resolutions achieved by each method are shown in Table 1 ▸. The best resolution was obtained when we applied *deepEMhancer*. However, this is a global value. Since in these tests all projections were generated from a computer-simulated map starting from a defined structural model, this model was used to quantitatively study how much of the new reconstructed map fits the ‘correct’ model. The *Q*-score (Pintilie *et al.*, 2020 ▸) and FSC-Q (Ramírez-Aportela *et al.*, 2021 ▸) methods were applied, which perform the calculations locally. As shown in Table 1 ▸, the best results were obtained with *deepEMhancer*. Additionally, Fig. S2 shows the map obtained using *deepEMhancer* superimposed on the starting atomic model, where the high level of agreement can be appreciated. Note these results indicate that the reconstructed map which best represents the ground truth model is the one obtained using *deepEMhancer*, indicating our proposed method has not introduced systematic artifacts in the maps.

Further analysis on the control of the appearance of artifacts was conducted by filtering the particles at different resolutions and varying the noise levels before refinement. First the particles were low-pass filtered at frequencies of 3, 5 and 8 Å with a raised cosine of 0.0064 (in discrete frequency normalized to 0.5), while in all cases Gaussian noise was added with zero mean and 150 standard deviation (SD). This implies that the data generated do not present frequencies above approximately 2.92, 4.76 and 7.40 Å. Fig. S3 shows the behavior of the FSC curve for the different reconstructions using standard *RELION* or in combination with *deepEMhancer*. The resolution achieved using *deepEMhancer* was slightly higher in all cases than using standard *RELION*, but in no case did the resolution exceed the cutoff frequency.

The dependency on the level of noise was tested in a second experiment in which different levels of Gaussian noise were added (with 50, 200, 400 and 1000 SD; Fig. S1) to the set of particles filtered to 5 Å. Fig. S4 shows the FSC curves for the reconstructed maps. In the case of a very high noise level (corresponding to the case of noise with 1000 SD), the *HEALPIX* variable does not exceed 2, so in this case *deepEMhancer* is not applied in any iteration and the resolution achieved is the one of standard *RELION* (17 Å; data not shown in the supporting figures). In Fig. S4 we observe that, with increasing noise, the resolutions achieved using standard *RELION* decrease, whereas this effect is less accentuated in the case of introducing *deepEMhancer*.

**Figure 1:**
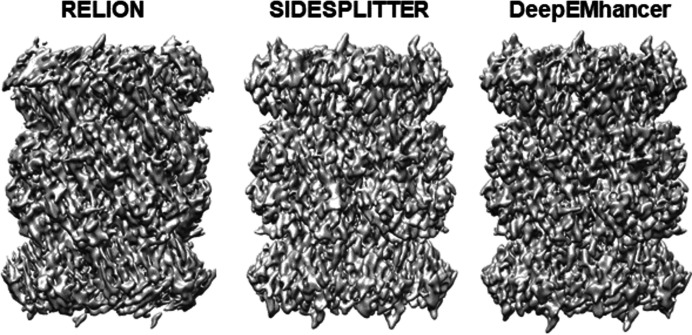
Refinement of synthetic data generated from the 20S proteasome structure (PDB entry 6bdf). Three different refinements were made using standard *RELION*, using *SIDESPLITTER* or incorporating *deepEMhancer*.

The second case corresponds to the known structure of the 20S proteasome (PDB entry 6bdf). In this test, the set of projections generated was not homogeneous, simulating the occurrence of preferred directions. Some previous work has shown an ‘attraction’ problem in *RELION* when the data have an over-abundance of projections in certain directions (Sorzano *et al.*, 2021 ▸, 2010 ▸). This test was designed to allow us to study the effect of *deepEMhancer* when there were important differences in the number of images along the different projection directions. Using these data, the resolutions obtained in the different reconstructions were similar (Table 2 ▸). However, we can observe that the density map obtained using *deepEMhancer* is better than those obtained using standard *RELION* and *SIDESPLITTER* (Fig. 1 ▸). In particular, the map obtained by *RELION* shows a higher deformation, a consequence of a severe preferential direction problem. *deepEMhancer* improves the local alignment of *relion_refine* and corrects, to some extent, the attraction when there are preferred directions in the sample. This improvement is also remarkable regarding *Q*-score and FSC-Q results (Table 2 ▸).

Our next experiment aims to check whether our algorithm works incorrectly in the presence of a very low signal-to-noise ratio. For that purpose, we simulated 18 309 noise-only images and reconstructed them using icosahedral symmetry, with a virus (EMD-23321) as the initial volume (this is one of the worst possible cases for processing, as reconstruction artifacts can be easily reinforced). We observed that our algorithm was never applied because *RELION* did not enter into the local refinement step due to the low resolution of the reconstructed map.

Finally, we checked the possible presence of artifacts during refinement using projections generated from the map reconstructed with the EMPIAR-10391 dataset, but in this case adding them to ‘pure noise’ particles obtained from the deposited micrographs (see Methods[Sec sec2]). This experiment is closer to a real experiment. The projections were low-pass filtered at frequencies of 3, 5 and 8 Å with a raised cosine of 0.0064 (in discrete frequency normalized to 0.5). To test different signal-to-noise ratios, the noise levels were increased by 5, 10 and 15×, respectively, before the addition of the generated projections. Thus, different datasets were created, with the projections filtered at varying resolutions and using three levels of noise. These datasets were used for refinement using standard *RELION* and *deepEMhancer*. Figs. S5 and S6 show the FSC curves obtained from the refinement using noise levels increased by 5 and 10×, respectively. As shown in the figures, in no case were resolutions higher than the expected thresholds obtained due to the filtering of the generated projections. In the dataset reconstructions with noise increased by 15×, the *HEALPIX* variable did not exceed 2, so *deepEMhancer* was not applied.

### 3.2. Results on experimental datasets

Having verified the advantages of using *deepEMhancer* in reconstructions with simulated data, we tested its applicability with experimental data. Three datasets were obtained from *EMPIAR*: EMPIAR-10391, EMPIAR-10254 and EMPIAR-10420. The cases studied correspond to membrane proteins, because this class of structure is the most likely to benefit from regularizers that consider the spatial non-uniformity of the reconstructions.

#### 3.2.1. Structure of arabino­furan­osyltransferase

**Figure 2:**
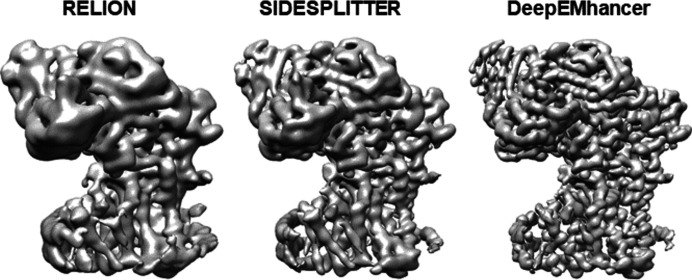
Refinements of 37 814 particle images of AftD in lipid nanodiscs (EMPIAR-10391). Comparison of refinement results using standard *RELION*, *SIDESPLITTER* and *deepEMhancer*. No local filtering or sharpening operations were used and the threshold is set to keep the enclosed volume constant.

In a first study using experimental data, the dataset of arabino­furan­o­syltransferase (AftD) (EMPIAR-10391) was used. AftD contains a membrane-embedded portion with 16 transmembrane helices and a soluble periplasmic portion (Hoffmann *et al.*, 2008 ▸). A total of 37 814 particle images of AftD in lipid nanodiscs were used for 3D reconstruction in *RELION*. For comparison, three reconstructions were performed using standard *relion_refine*, *SIDESPLITTER* and *deepEMhancer*. Figs. 2 ▸ and S9 show the density maps obtained by each method. Though the map obtained with *SIDESPLITTER* is higher quality than that obtained with standard *RELION*, the best quality map was obtained using *deepEMhancer*. Better definition is observed in both the transmembrane helices and the soluble portion. This map achieves a resolution of 2.83 Å by the gold-standard FSC of 0.143 Å, whereas 4.24 Å was obtained with standard *RELION* and 3.99 Å with *SIDESPLITTER* [Fig. S7(*a*)]. The improvement is also reflected in the local resolution histograms determined by *deepRes* (Ramírez-Aportela *et al.*, 2019 ▸) [Fig. S8(*a*)]. The median local resolution was 4.12 Å using *deepEMhancer*, 4.96 Å with *SIDESPLITTER* and 5.96 Å with standard *RELION*. We note that there is no additional sharpening operation involved in any of the three results.

An interesting practical question might be posed at this stage about whether the differences presented in these analyses might indeed appear should postprocessing of all the different maps by *deepEMhancer* be done consistently at the end of the reconstruction process instead of inside the iteration loop. The answer to this question is given in Figs. S11 and S12. Indeed, the differences are less pronounced than with the protocol previously presented, however, we still find areas of lower resolution where applying *deepEMhancer* within *RELION* offers significant advantages over using it for postprocessing. Fig. S11(*a*) shows a fragment of the reconstruction using *deepEMhancer* within *RELION*, while panels (*b*), (*c*) and (*d*) show the results after applying *deepEMhancer* as postprocessing on the maps obtained using standard *RELION*, or in combination with *SIDESPLITTER* or *deepEMhancer*, respectively. In this figure we can see different areas (indicated in red) where better performance is obtained by combining *RELION* with *deepEMhancer*. Additionally, and using the multimethod integrative capabilities of *Scipion*, we can explore this question further and consider the actual angular differences introduced to the refinement process when *deepEMhancer* is used as an integral part of the iterative process. Fig. S12 compares the differences in spatial shifts (Å) and angular alignment (°) when using three different workflows that are presented throughout this work (the differences between *RELION* and *SIDESPLITTER* are shown in dark blue, and the differences between *RELION* and *deepEMhancer* are shown in cyan). The differences are to be understood in the context that we are in the same local minima of the optimization process (*i.e.* they cannot be very large), but they clearly and systematically occur, indicating that, indeed, the iterative use of *deepEMhancer* has impacted the very essence of the refinement process, as it is the finding of the projection geometry.

#### 3.2.2. Structure of TRPV5

**Figure 3:**
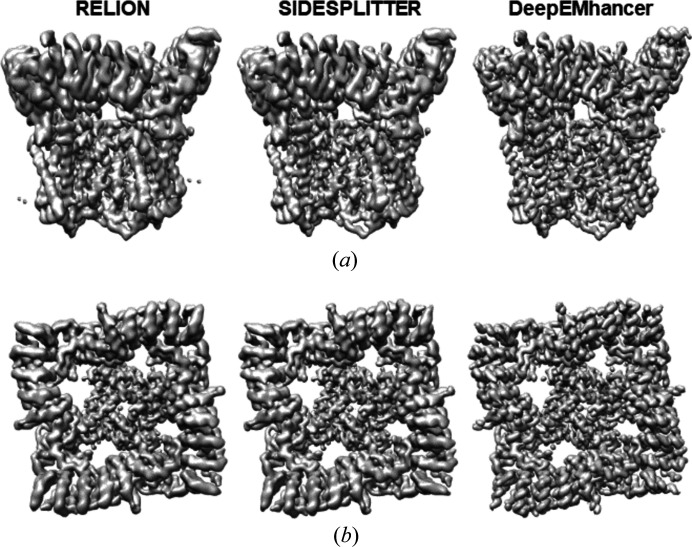
Refinements of 87 603 particle images of TRPV5 in lipid nanodiscs (EMPIAR-10254). Comparison of refinement results using standard *RELION*, *SIDESPLITTER* and *deepEMhancer*. No local filtering or sharpening operations were used and the threshold was set to keep the enclosed volume constant. (*a*) Side view and (*b*) top view of the reconstructions.

The second case corresponds to the structure of full-length TRPV5 in lipid nanodiscs (EMPIAR-10254). In this case, 87 603 previously deposited particles were used for the reconstructions using *C*4 symmetry. The three workflows previously described using *relion_refine* algorithms were run and their corresponding 3D reconstructions are depicted in Figs. 3 ▸ and S10. As can be seen, the map obtained with *deepEMhancer* appears to be better quality, with a clearer definition of the helical pitch in both the region immersed inside the nanodisc and the intracellular domains. *deepEMhancer* reduces the influence of noise on alignment and produces an improvement in resolution of the protein. Using the gold standard FSC, resolutions of 3.39, 3.35 and 2.15 Å were achieved for *RELION*, *SIDESPLITTER* and *deepEMhancer*, respectively [Fig. S7(*b*)]. The median local resolution of *deepRes* histograms [Fig. S8(*b*)] was 5.04 Å for *RELION*, 4.58 Å for *SIDESPLITTER* and 3.41 Å for *deepEMhancer*.

#### 3.2.3. Structure of arabinosyltransferase B

**Figure 4:**
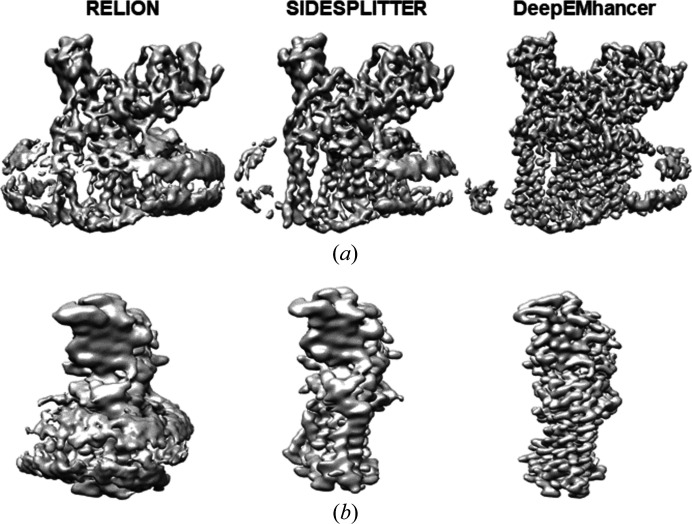
Refinements of 57 970 particle images of EmbB in lipid nanodiscs (EMPIAR-10420). Comparison of refinement results using standard *RELION*, *SIDESPLITTER* and *deepEMhancer*. No local filtering or sharpening operations were used and the threshold was set to keep the enclosed volume constant. (*a*) Side view and (*b*) alternative side view of the reconstructions.

The third dataset pertains to the structure of the arabinosyltransferase B (EmbB), a 117 kDa integral membrane enzyme consisting of 11 transmembrane helices and 2 distinct periplasmic carbohydrate binding modules (CBMs). The dataset obtained from EMPIAR-10420 contains 57 970 previously processed particles that were used for the 3D reconstructions. Fig. 4 ▸ shows the maps obtained, which reach resolutions of 4.0 (*RELION*), 3.66 (*SIDESPLITTER*) and 2.69 Å (*deepEMhancer*) [Fig. S7(*c*)]. However, the reconstructed maps present elongations, which are typical of particles with preferred orientations/directions. As discussed previously in the proteasome test, when performing 3D angular assignment using *RELION*, some directions might attract particles from other directions, most likely nearby directions. This problem is accentuated in the *RELION*-only map, although it is also very evident with *SIDESPLITTER*. Using *deepEMhancer* we can see that, although the problem persists, the map obtained shows a remarkable correction of this effect. *deepEMhancer* allows better handling of anisotropic SNR.

## 4. Discussion

Extreme noise in the images acquired from the microscope may hinder the correct angular assignment of particles during 3D reconstruction in cryo-EM. This issue is even more complex for integral membrane proteins, which are embedded in detergent micelles or lipid nanodiscs and exhibit greater disorder and flexibility. These characteristics increase the spatial variability in the reconstructions, which is reflected in greater SNR heterogeneity. However, traditional reconstruction algorithms in cryo-EM assume spatial homogeneity and the regularizers used to avoid overfitting have also routinely been spatially invariant. Nonetheless, applying a shift-invariant filter may cause noise accumulation in some areas, while in others the signal may be degraded.

Recently, adaptive regularizers have been introduced in reconstruction methods (Punjani *et al.*, 2020 ▸; Ramlaul *et al.*, 2020 ▸). These regularizers consider the differences in spatial SNR and help to mitigate overfitting. The new *RELION* functionality (Kimanius *et al.*, 2021 ▸) that allows the use of external proposals in the refinement (previously used by *SIDESPLITTER*) is a very important tool for the development of new hybrid reconstruction methods. This article shows the benefits of integrating *deepEMhancer* within *relion_refine*, helping to mitigate overfitting and obtain better quality 3D reconstructions. *deepEMhancer* introduces information from the macromolecules already learned during the previous training and has a double effect on the intermediate reconstructions; on the one hand, it has a masking/denoising effect, while at the same time enhancing the signal. Furthermore, *deepEMhancer* takes into account the anisotropic SNRs and improves angular assignment in cases characterized by preferred directions.

Note that *deepEMhancer* is only applied during local angular assignment in the last iterations of the refinement. This allows the possibility of applying other regularizers during global assignment, such as *SIDESPLITTER*. However, we found no apparent benefit with the combination of *SIDESPLITTER* and *deepEMhancer* in the cases tested. The combination of different regularizers can be evaluated in more detail in future work.

## Supplementary Material

Supporting figures. DOI: 10.1107/S2052252522006959/fq5020sup1.pdf

## Figures and Tables

**Table 1:** Validation metrics for different reconstructions of β-galactosidase FSC resolution first, followed by two map-to-model validation criteria.

	*RELION*	*SIDESPLITTER*	*deepEMhancer*
Resolution (Å)	3.12	3.03	2.87
*Q* scores	0.772	0.780	0.789
FSC-Q (Å)	0.37	0.37	0.33

**Table 2:** Validation metrics for different reconstructions of 20S proteasome FSC resolution first, followed by two map-to-model validation criteria.

	*RELION*	*SIDESPLITTER*	*deepEMhancer*
Resolution (Å)	4.0	4.0	4.0
*Q* scores	0.284	0.410	0.481
FSC-Q (Å)	4.06	2.01	0.75
