# Supplementary material for: Higher resolution in cryo-EM by the combination of macromolecular prior knowledge and image-processing tools
Source: IUCrJ. 2022 Aug 3;9(Pt 5):632–8. doi: 10.1107/S2052252522006959 (PMC9438491; doi:10.1107/S2052252522006959)
Supplement: Supplementary file 1 [file m-09-00632-sup1.pdf]

IUCrJ

Volume 9 (2022)

Supporting information for article:

Higher resolution in cryo-EM by the combination of macromolecular prior knowledge and image processing tools

Erney Ramírez-Aportela, Jose M. Carazo and Carlos Oscar S. Sorzano

Supplementary Information

Higher resolution in Cryo-EM by the combination of macromolecular prior knowledge and image processing tools.

Erney Ramírez-Aportela*[a], Jose Maria Carazo*[a], Carlos Oscar S. Sorzano*[a,b]

[a]Biocomputing Unit, National Center for Biotechnology (CSIC), Darwin 3, Campus Univ. Autónoma de Madrid, 28049 Cantoblanco, Madrid, Spain.

[b]Univ. CEU San Pablo, Campus Urb. Montepríncipe, Boadilla del Monte, 28668 Madrid, Spain.

*Corresponding authors: erney.ramirez@gmail.com, carazo@cnb.csic.es, coss@cnb.csic.es

Supplementary Figure 1

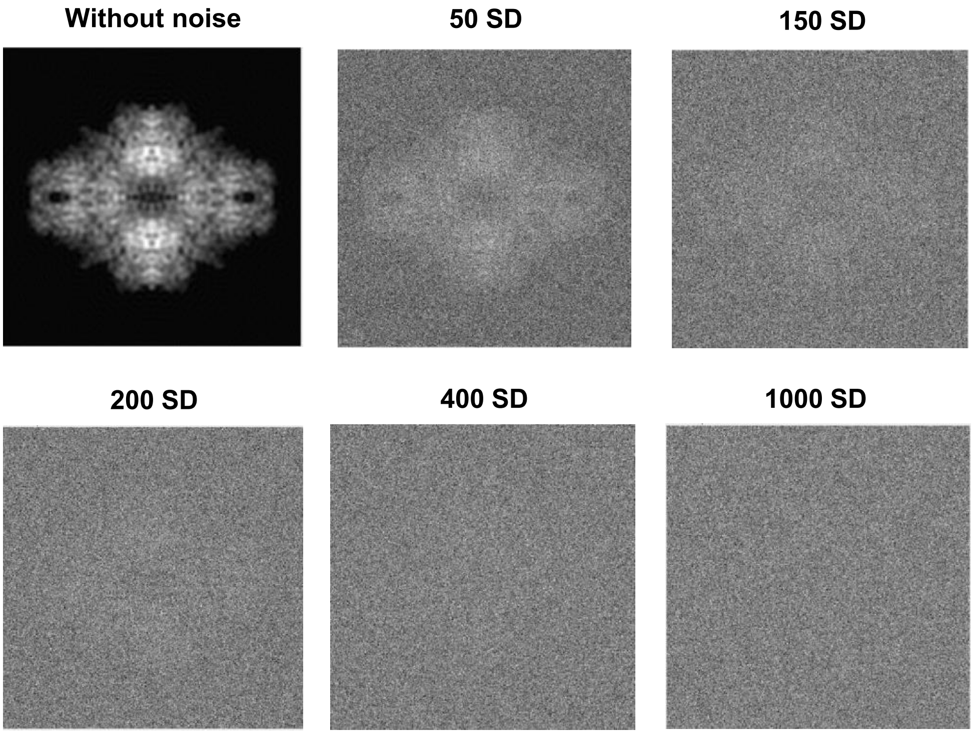

Supplementary Figure 1. Example of simulated ß-galactosidase images used in the refinements. The images are shown with the different levels of Gaussian noise added. The first panel shows the original image without noise, followed by the images with noise of mean 0 and standard deviation (SD) of 50, 150, 200, 400 and 1000, respectively.

Supplementary Figure 2

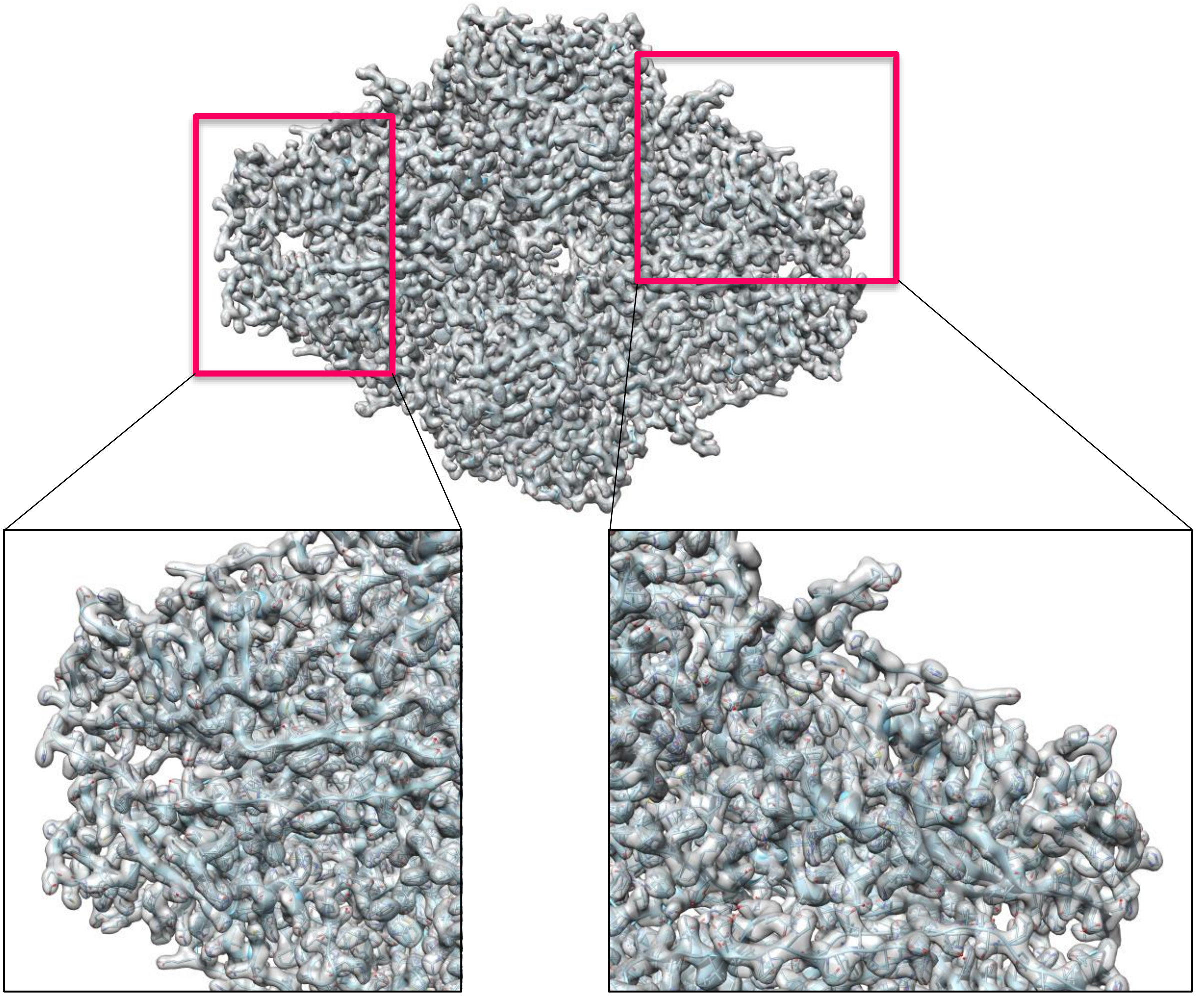

Supplementary Figure 2. Map obtained using deepEMhancer with the set of particles simulated using the atomic model of ß-galactosidase (pdb id: 3j7h). The map is shown superimposed on the atomic model used in the generation of the starting data. Two zoomed areas are highlighted at the bottom.

Supplementary Figure 3

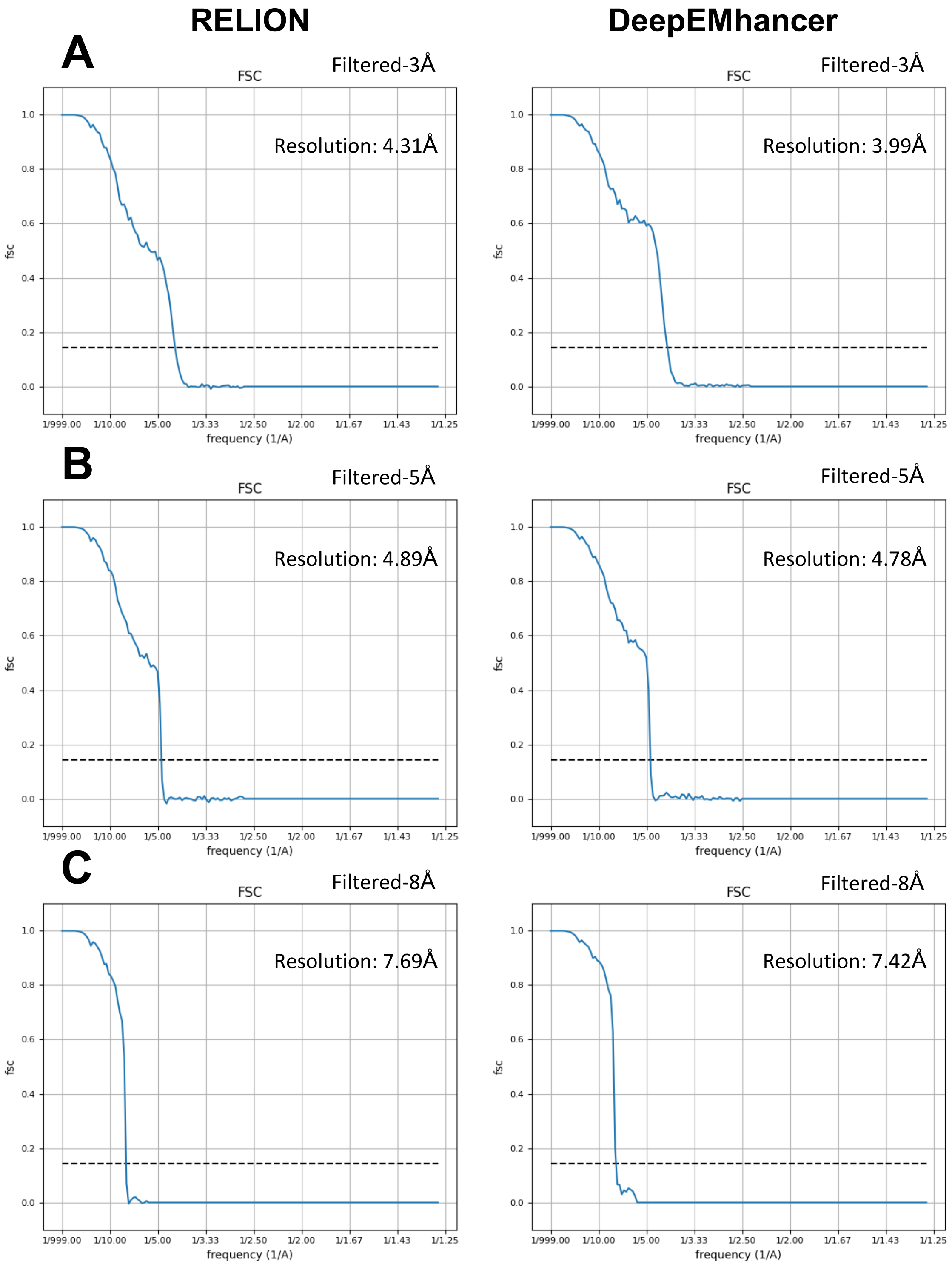

Supplementary Figure 3. FSC curves obtained for reconstructed maps using simulated data. The maps were reconstructed using standard RELION (shown on the left) or by introducing deepEMhancer (shown on the right). The particles used for the reconstructions were low-pass filtered at different resolutions of 3 Å (panel A), 5 Å (panel B) and 8 Å (panel C) with raised cosine filter of 0.0064 (in normalized units). Gaussian noise with zero mean and 150 SD was used in every case. The resolution achieved in each case is shown within each chart.

Supplementary Figure 4

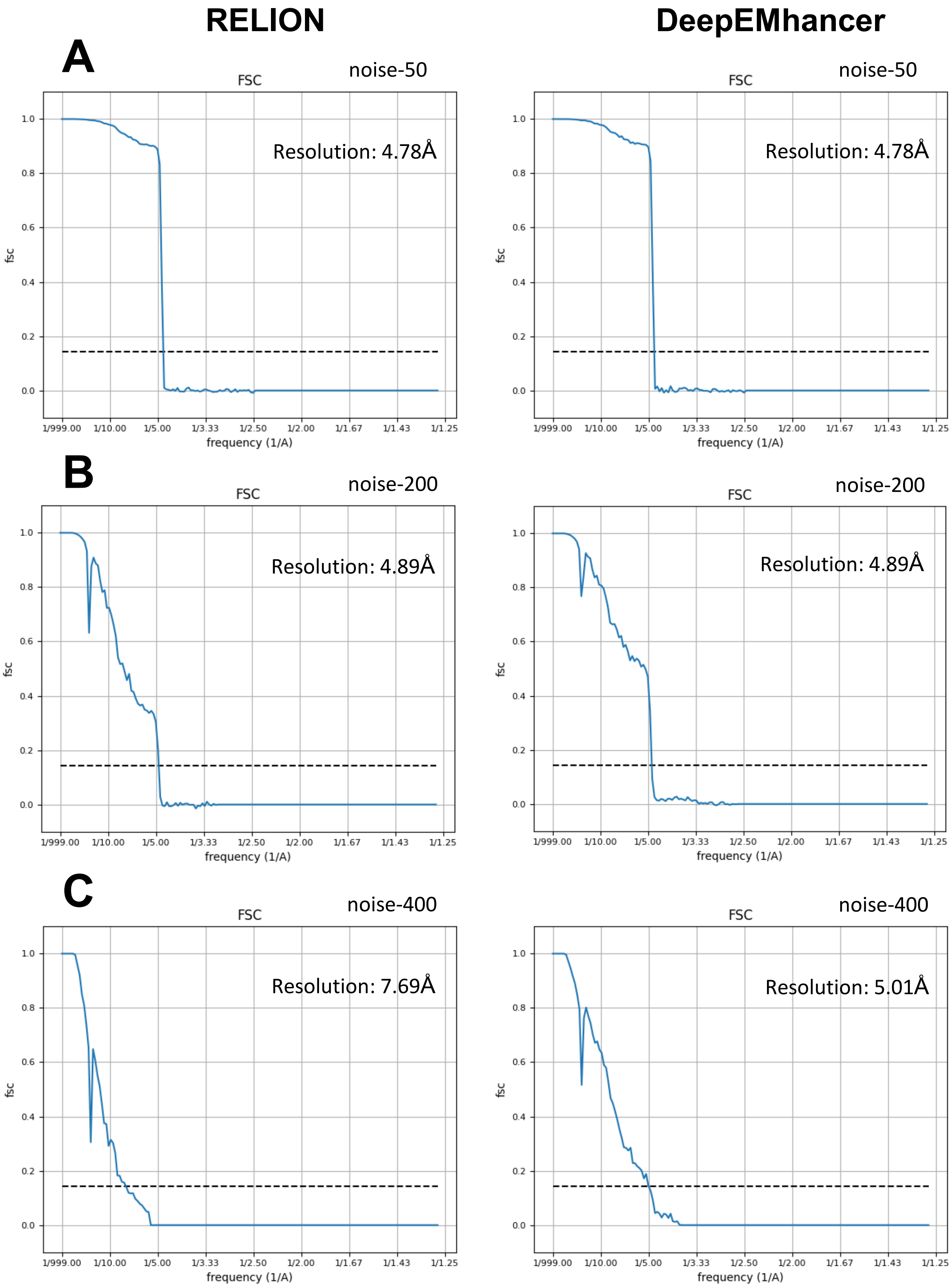

Supplementary Figure 4. FSC curves obtained for reconstructed maps using simulated data. The maps were reconstructed using standard RELION (shown on the left) or by introducing deepEMhancer (shown on the right). The particles used for the reconstructions were low-pass filtered at 5 Å with a raised cosine filter of 0.0064 (in normalized units) and different levels of Gaussian noise were added. (A) Gaussian noise with zero mean and 50 SD was added to the particles used, (B) Gaussian noise with zero mean and 200 SD and (C) Gaussian noise with zero mean and 400 SD was used. The resolution achieved in each case is shown within each chart.

Supplementary Figure 5

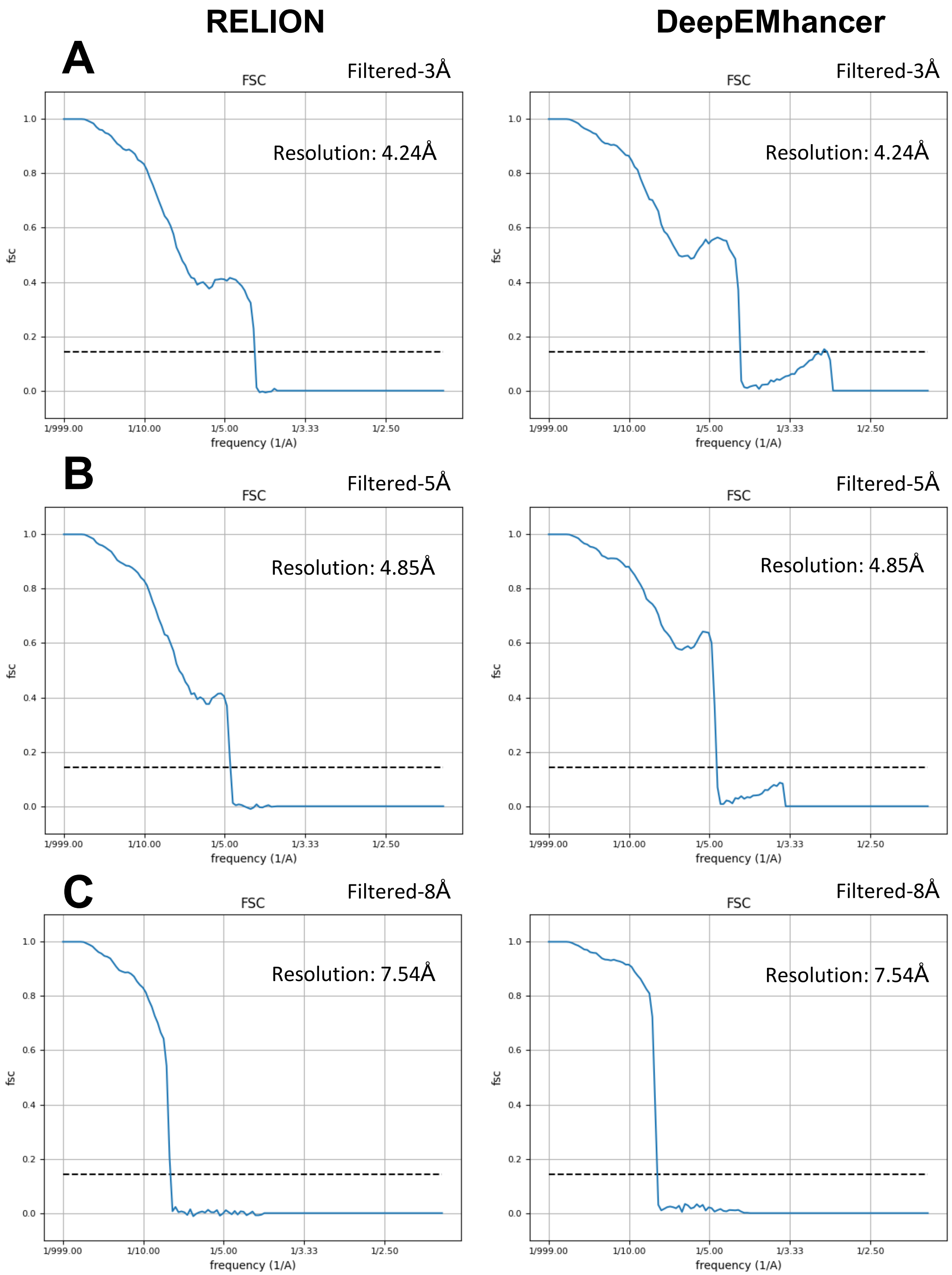

Supplementary Figure 5. FSC curves obtained for reconstructed maps using projections added to pure noise particles multiplied by a factor of 5. The maps were reconstructed using standard RELION (shown on the left) or by introducing deepEMhancer (shown on the right). The projections used for the reconstructions were low-pass filtered at different resolutions of 3 Å (panel A), 5 Å (panel B) and 8 Å (panel C) with raised cosine filter of 0.0064 (in normalized units). The noise of the picked particles was increased by a factor of 5.

Supplementary Figure 6

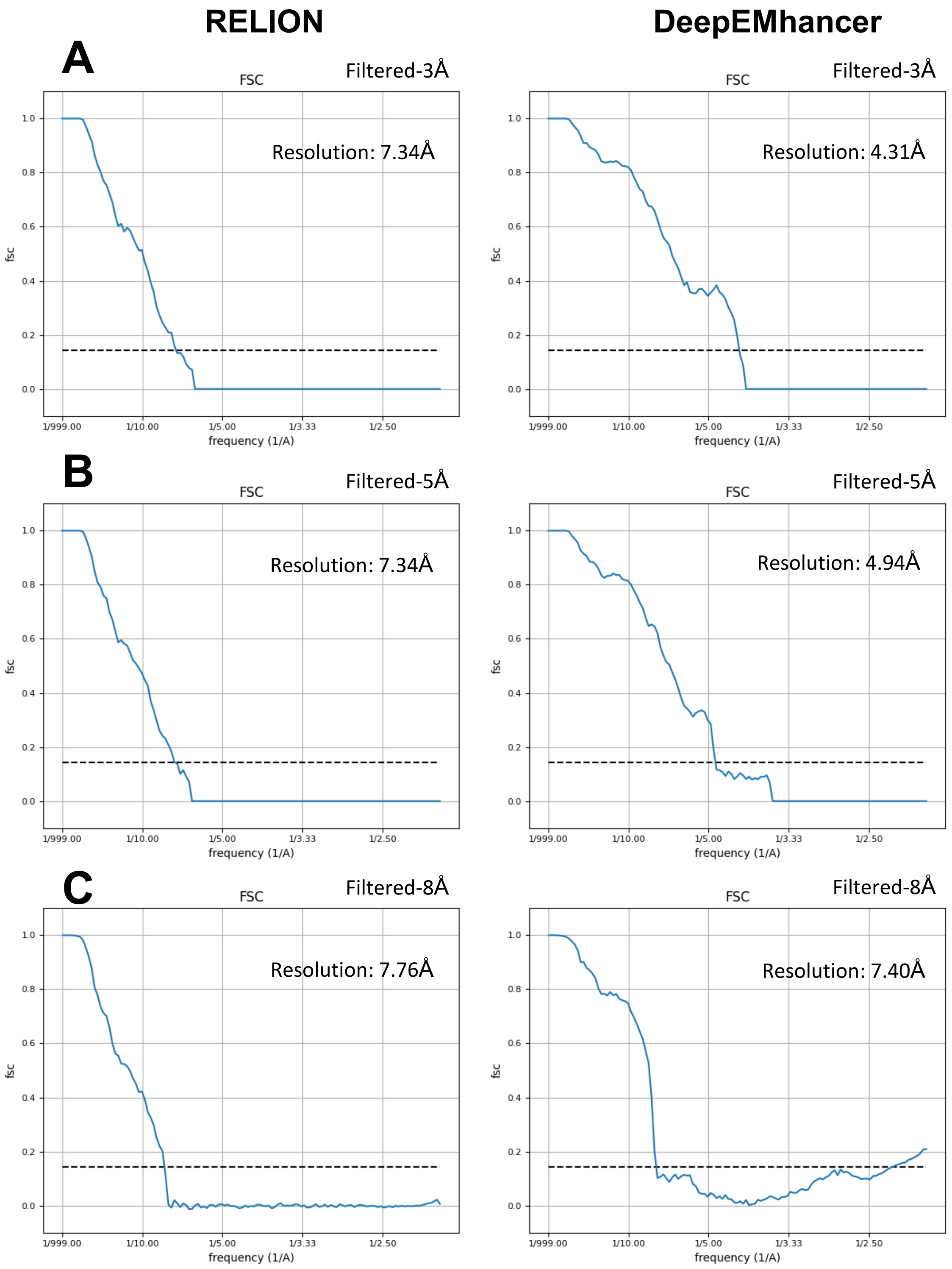

Supplementary Figure 6. FSC curves obtained for reconstructed maps using projections added to pure noise particles multiplied by a factor of 10. The maps were reconstructed using standard RELION (shown on the left) or by introducing deepEMhancer (shown on the right). The projections used for the reconstructions were low-pass filtered at different resolutions of 3 Å (panel A), 5 Å (panel B) and 8 Å (panel C) with raised cosine filter of 0.0064 (in normalized units). The noise of the picked particles was increased by a factor of 10.

Supplementary Figure 7

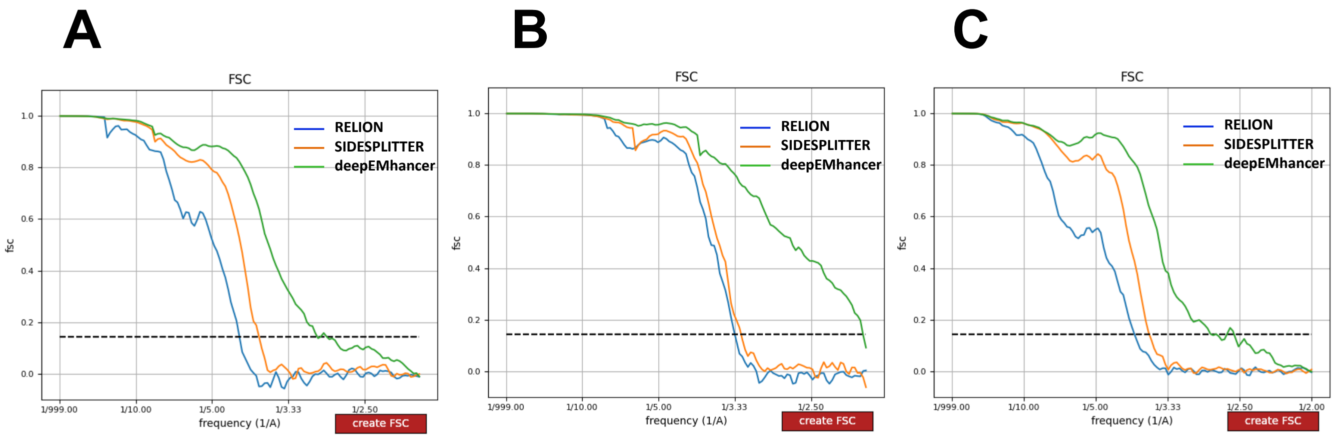

Supplementary Figure 7. Comparison of FSC curves obtained for reconstructed maps with different experimental dataset when using standard RELION, SIDESPLITTER or deepEMhancer. (A) The dataset of Arabinofuranosyltransferase AftD (EMPIAR-10391) was used for reconstruction. (B) The full length TRPV5 in lipid nanodiscs dataset (EMPIAR-10254) was employed in this case. (C) Images from Arabinosyltransferase B (EmbB) (EMPIAR-10420) were used for this test.

Supplementary Figure 8

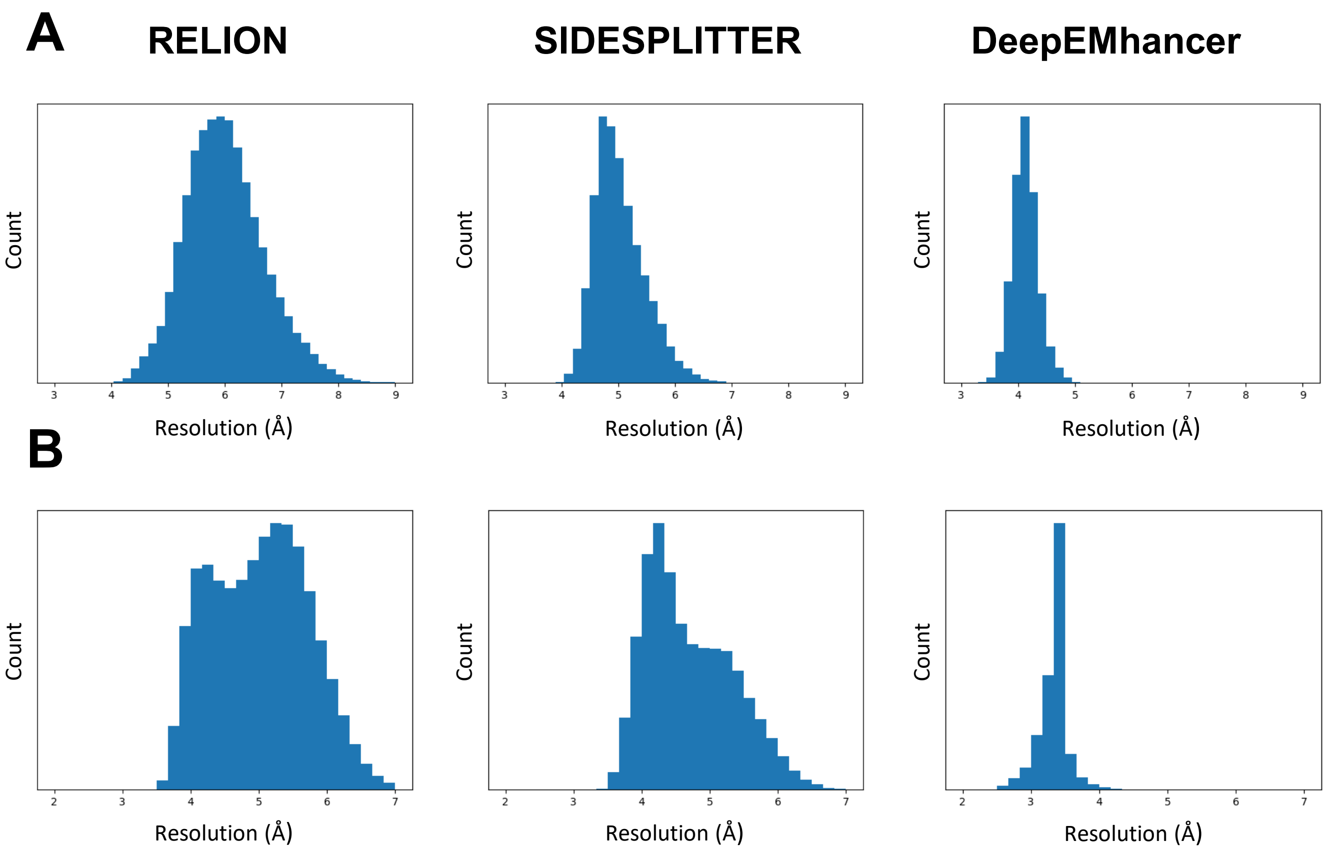

Supplementary Figure 8. Local resolution results for reconstructed maps. The resolution histograms obtained with deepRes for the maps reconstructed by the different methods (RELION, SIDESPLITTER and deepEMhancer) are shown for (A) AftD (EMPIAR-10391), (B) TRPV5 (EMPIAR-10254).

Supplementary Figure 9

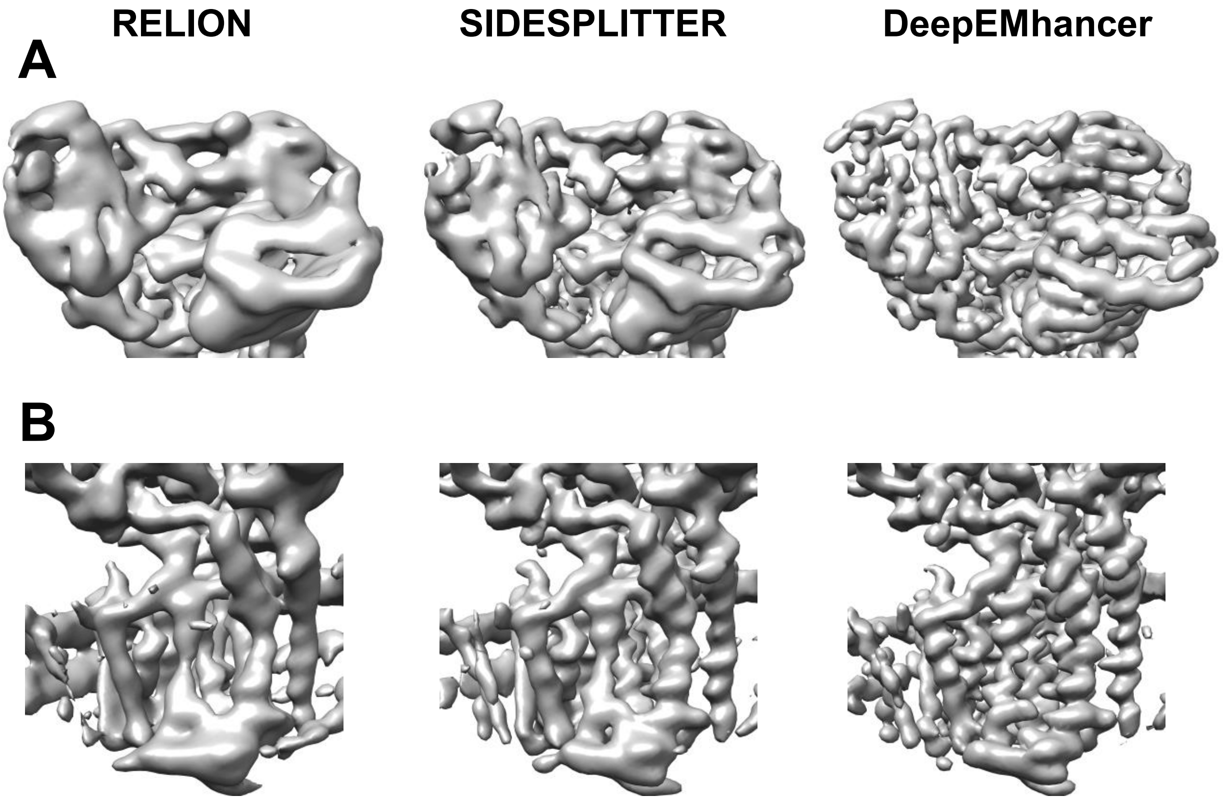

Supplementary Figure 9. Refinements of 37,814 particle images of AftD in lipid nanodiscs (EMPIAR-10391). Comparison of refinement results using standard RELION, SIDESPLITTER and deepEMhancer. (A) A close-up is shown in the soluble periplasmic portion. (B) The transmembrane region with the highest resolution is zoomed in. No local filtering or sharpening operations are used and the threshold are set to keep the enclosed volume constant.

Supplementary Figure 10

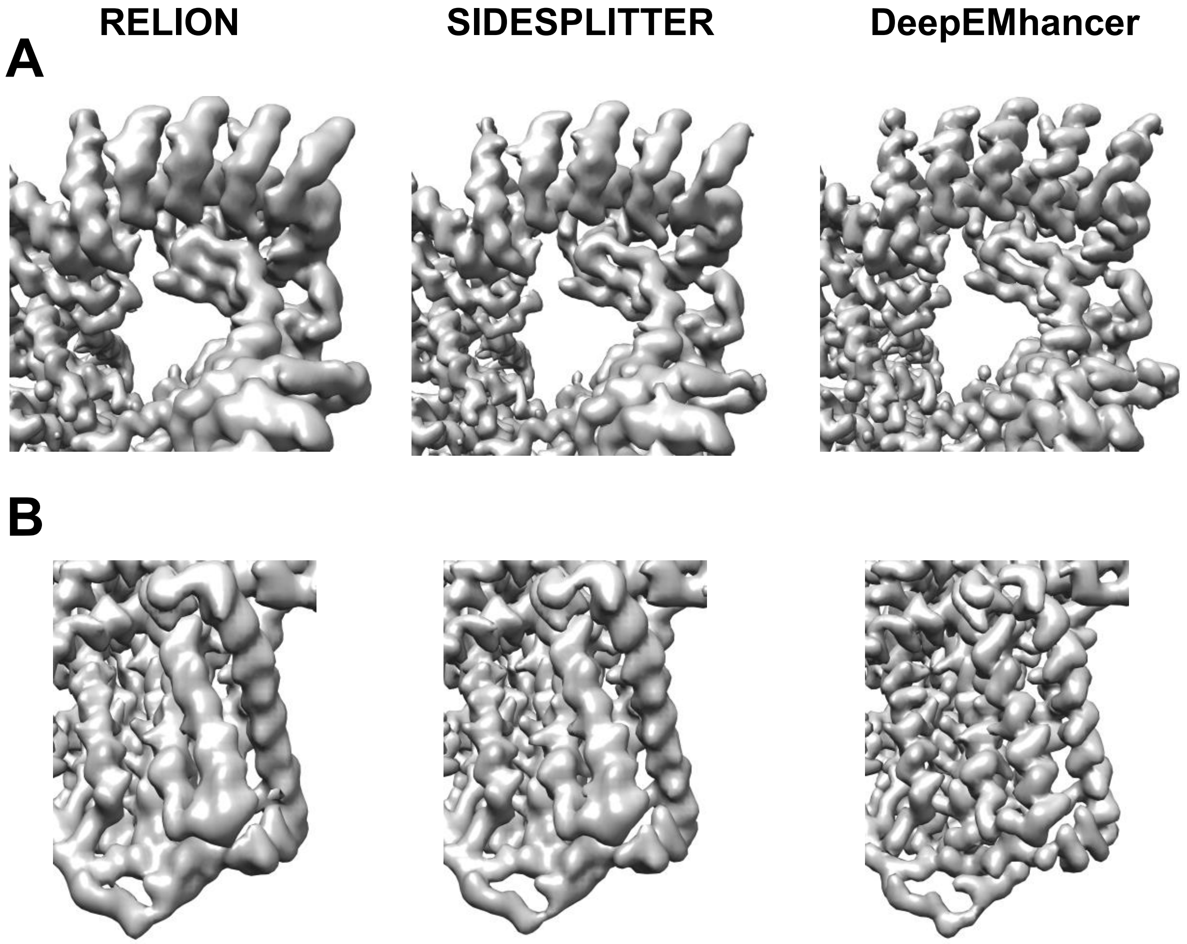

Supplementary Figure 10. Refinements of 87,603 particle images of TRPV5 in lipid nanodiscs (EMPIAR-10254). Comparison of refinement results using standard RELION, SIDESPLITTER and deepEMhancer. (A) The extracellular domain area is amplified. (B) The transmembrane region is zoomed in. No local filtering or sharpening operations are used and the threshold are set to keep the enclosed volume constant.

Supplementary Figure 11

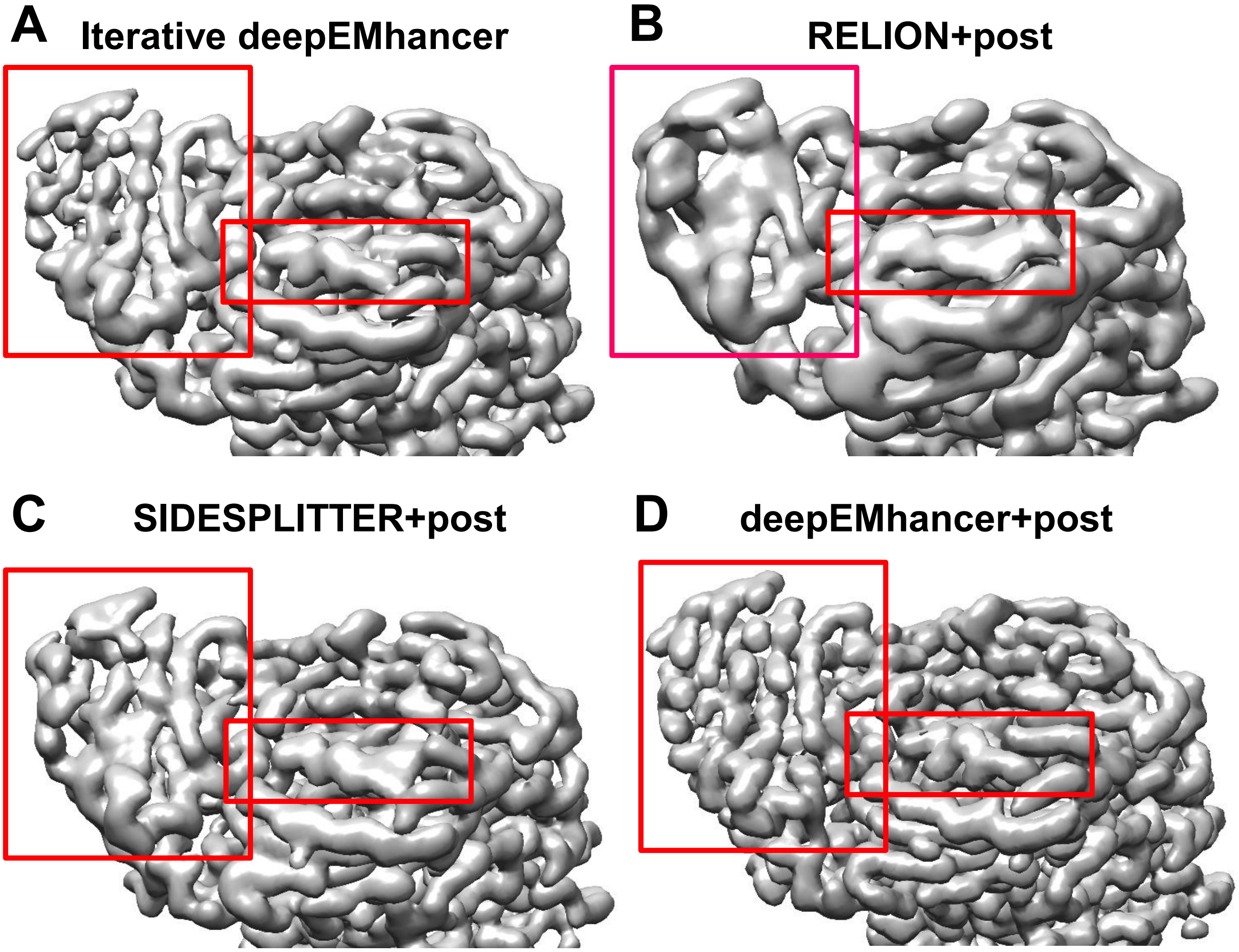

Supplementary Figure 11. Fragments of the maps reconstructed by the different methods (RELION, SIDESPLITTER and deepEMhancer) using the datasef of Arabinofuranosyltransferase AftD (EMPIAR-10391), after applying deepEMhancer as postprocessing. (A) The map resulting from applying deepEMhancer within the iterative process, without applying any postprocessing operation, is shown. (B) The fragment shown corresponds to the map obtained using standard RELION and applying deepEMhancer as final postprocessing. (C) The fragment corresponds to the map obtained using SIDESPLITTER and using deepEMhancer as postprocessing. (D) The fragment corresponds to the map obtained using RELION with deepEMhancer and postprocessed with deepEMhancer.

Supplementary Figure 12

A

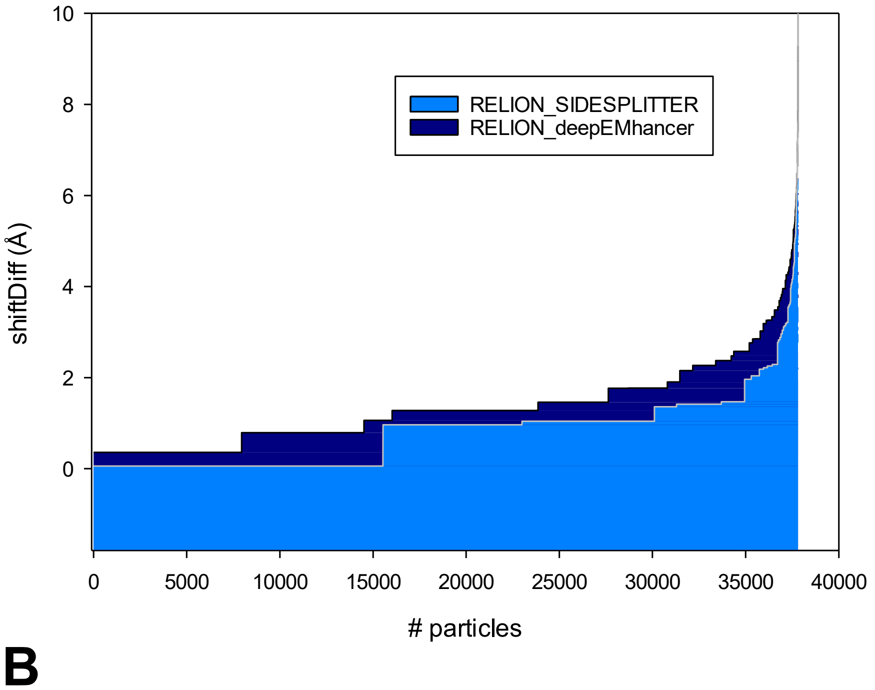

B

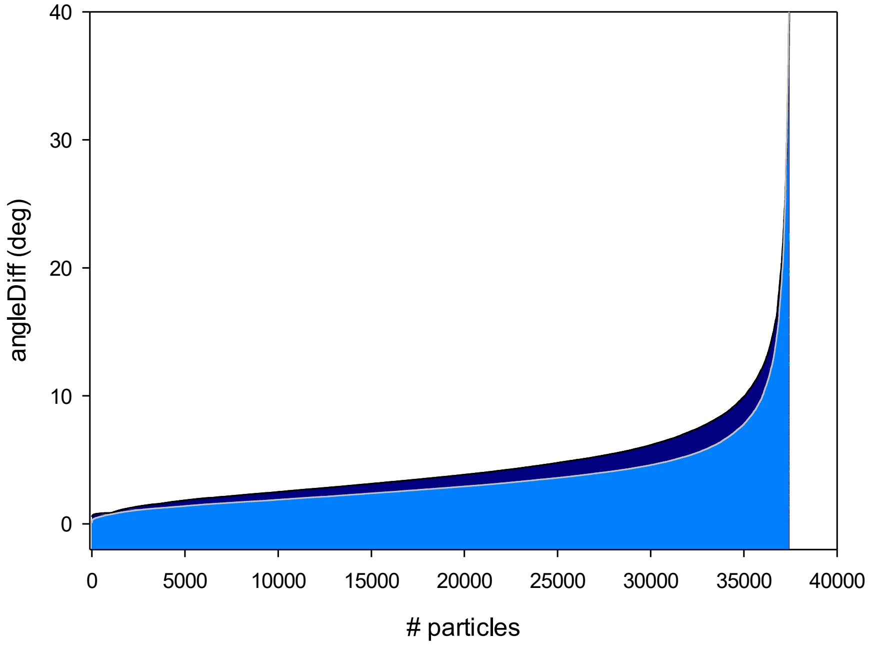

Supplementary Figure 12. Differences in particles alignment pose and shift between different refinement approaches using the datasef of Arabinofuranosyltransferase AftD (EMPIAR-10391). (A) The differences in particle shift between the methods are shown. In dark blue are the differences between applying the standard RELION or SIDESPLITTER and in cyan the differences between applying the standard RELION or deepEMhancer. (B) Differences in angular distribution are shown.
